# Identifying possible risk factors for cesarean scar pregnancy based on a retrospective study of 291 cases

**DOI:** 10.1111/jog.14163

**Published:** 2020-01-14

**Authors:** XianYi Zhou, Hua Li, XiaoDong Fu

**Affiliations:** ^1^ Department of Gynecology and Obstetrics the First Affiliated Hospital of Southwest Medical University Luzhou China; ^2^ Department of Gynecology and Obstetrics Chengdu Jin Jiang Hospital for Women and Children Health Chengdu China

**Keywords:** cesarean scar defect, cesarean scar pregnancy, cesarean section, clinical characteristics, risk factors

## Abstract

**Aim:**

Cesarean scar pregnancy (CSP) is a rare but life‐threatening type of ectopic pregnancy. This study's aim is to investigate the clinical characteristics and possible risk factors for cesarean scar pregnancy.

**Methods:**

A clinically randomized, unpaired and retrospective case–control study was implemented. A study group of 291 CSP patients and a control group of 317 full‐term pregnant women with a history of cesarean section (CS) were recruited in our hospital from May 2013 to October 2018. Their demographic characteristics and medical and obstetric history were collected.

**Results:**

Only symptoms suggestive of an impending abortion, such as vaginal bleeding with or without abdominal pain, were identified as the clinical characteristics of CSP. Maternal age older than 35 years, gravidity higher than 3 (especially gravidity higher than 5), more than two induced abortions (especially more than five abortions), an interval of less than 5 years (especially less than 2 years) between the current pregnancy and the last CS, history of CS performed in a rural hospital, history of induced abortions after CS and retroposition of the uterus were possible independent risk factors for CSP.

**Conclusion:**

CSP is a result of a combination of multiple factors associated with CS. There are no unique early clinical features of CSP. As a unique type of ectopic pregnancy, early diagnosis, early termination and early clearance should be the treatment principles. Further research is needed to evaluate the relationship between the cesarean scar defect and CSP in the future.

## Introduction

Cesarean scar pregnancy (CSP), which is a unique type of ectopic pregnancy, was first defined in 1978.^1^ CSP is a late serious complication of cesarean section (CS) that is defined as a gestational sac implanted in the scar from a previous CS. The incidence of CSP is reported to be 1:2226–1:1800, occurring in 1.15% of women with previous cesarean deliveries and representing 6.1% of all ectopic pregnancies after cesarean deliveries.^2^, ^3^ Currently, the incidence of CSP is increasing rapidly because of the increase in the CS rate^4^ and the widespread use of transvaginal sonography.^5^ At present, there are many reports about the etiology and pathogenesis of CSP at home and abroad, but the exact etiology is still not completely clear. The relationship between cesarean scar defects and CSP has remains unclear. There are no specific early clinical manifestations of CSP, but this ectopic pregnancy can cause serious complications, such as placental implantation, uterine rupture and uncontrolled hemorrhage, infertility or even loss of life.^6^, ^7^ Due to the rarity of CSP, there is no consensus on the treatment and management, and individualized therapy should be performed. In this study, we present the patients' demographic characteristics and medical and obstetric history based on our clinical experience with 291 CSP cases over 5 years in our hospital to investigate the clinical characteristics and some possible risk factors for CSP to provide a better understanding of the disease.

## Methods

In this randomized, unpaired and retrospective study, 291 patients with CSP whose pregnancies were terminated by surgery at the Department of Obstetrics and Gynecology, the First Affiliated Hospital of Southwest Medical University between May 2013 and October 2018 were selected as the observational group, and 317 full‐term pregnant women with a history of CS and pregnancies terminated by surgery were selected as the control group. The previous cesarean section in both groups was the transection of the lower uterine segment. The study was approved and registered in the Ethics Committee of the Affiliated Hospital of Southwest Medical University (registration number: KY2019032). The ethics committee approved related screening and data collection from these subjects. All work was undertaken following the provisions of the Declaration of Helsinki. Informed consent was obtained from all individual participants included in the study. Our hospital is the largest obstetrics and gynecology medical treatment unit in the southwestern part of Sichuan province. We receive referrals from Yunnan province, Guizhou province and other places. Therefore, the cases were well balanced and representative. All CSP cases were confirmed by color Doppler ultrasound and/or postoperative pathology. The typical sonographic findings of CSP are as follows: (i) empty intrauterine cavity and cervix with no gestational sac observed; (ii) gestational sac implanted in the anterior inferior segment of the uterine muscle layer (equivalent to the previous incision site from CS in the uterus), with or without a fetal pole and the presence or absence of cardiac activity; (iii) interrupted continuity of the myometrium in the anterior uterine wall with an obviously thin or invisible myometrial layer between the gestational sac and the bladder; and (iv) Color Doppler Flow Imaging (CDFI) showing high‐speed and low‐obstruction blood flow signal around the gestational sac.^8^ The patients' medical information was obtained from our hospital's electronic medical record system. We collected the patients' demographic characteristics and medical and obstetric history. The risk factors analyzed included maternal age, gravidity, parity, number of induced abortions, number of previous CS procedures, an interval between the last CS and the current pregnancy, history of induced abortion after CS, hospital grade of previous CS procedures, position of uterus, and indications for the previous CS procedures.

### Statistical analysis

This study was performed as a case–control study. All the data were managed with SPSS 20.0 (Statistical Package for Social Sciences, IBM). All categorical variables are reported as frequencies (*n*) and composition ratios (%) and were compared with the χ^2^ test. Variables that were significant in the univariate analysis were included as independent variables in a binary logistic regression analysis. An odds radio (OR) >1 and a *P* value <0.05 were considered statistically significant.

## Results

### Clinical characteristics

Table 1 shows the clinical characteristics of the included patients when they were admitted to our hospital. Except for a history of menopause, 34.50% of CSP patients experienced vaginal bleeding, 3.40% experienced abdominal pain, 11.30% experienced bleeding and pain, and 38.10% were asymptomatic. Notably, 12% of patients were referred to our hospital because of failed dilatation and curettage or abortion performed at other hospitals. The most serious cases involved patients who experienced uncontrolled hemorrhage during or after dilatation and curettage or induced abortion.

**Table 1:** Clinical manifestations at admission

Manifestations	*n*	%
Vaginal bleeding	100	34.50%
Abdominal pain	10	3.40%
Bleeding and pain	33	11.30%
Asymptomatic	113	38.10%
Failed treatment at another hospital	35	12%
Total	291	100%

CS, cesarean section.

### Risk factor analysis for CSP

Table 2 shows the univariate analysis in the CSP group versus the control group. The maternal age between the two groups was significantly different (*P* = 0.006). Patients with CSP were more likely to be women of advanced reproductive age (age older than 35 years). The gravidity and parity in the two groups were significantly different (*P* < 0.001). More patients in the CSP group had gravidity higher than 5 and parity higher than 2 than in the control group. Significant differences in the number of previous CS procedures and the number of induced abortions were noted between the two groups (*P* < 0.001). Approximately 41.49% (121 of 291) CSP patients had two or more previous CS procedures, while approximately 25.81% (82 of 317) of non‐CSP patients had two or more previous CS procedures. Approximately 65.3% (190 of 291) of CSP patients had two or more induced abortions, while approximately 31.2% (99 of 317) of non‐CSP patients had two or more induced abortions. Interestingly, significantly more patients in the CSP group had a history of induced abortion after CS than in the control group (*P* < 0.001). In addition, in this study, we found that the interval between the current pregnancy and the last CS affected the occurrence of CSP (*P* < 0.001). Approximately 15.8% (46 of 317) and 6.3% (20 of 291) of patients in the CSP and control groups, respectively, had an interval of less than 2 years between the current pregnancy and the last CS. In this analysis, we noticed that the indications for previous CS also affected the occurrence CSP. Fifty‐five (17.4%) and 24 (8.2%) patients in the control and CSP groups, respectively, received postlabor CS. In addition, patients in the CSP group were more likely to have had a previous CS performed in a rural hospital than those in the control group (*P* < 0.001). Finally, we also found that CSP was more likely to occur in women with retroposition of the uterus (*P* < 0.001).

**Table 2:** Univariate analysis of the CSP group versus control group, *n* (%)

Variables	Category	Control		CSP		χ^2^	*P*
		*n*	%	*n*	%		
Maternal age (years)	<35	238	75.1%	189	64.9%	7.447	0.006
	≥35	79	24.9%	102	35.1%		
Gravidity (times)	<3	82	25.9%	18	6.2%	79.725	0.000
	3–5	202	63.7%	169	58.1%		
	>5	33	10.4%	104	35.7%		
Parity (times)	<2	222	70.0%	143	49.1%	27.597	0.000
	≥2	95	30.0%	148	50.9%		
CS (times)	1	235	74.1%	170	58.4%	20.737	0.000
	2	81	25.6%	112	38.5%		
	≥3	1	0.3%	9	3.1%		
Abortion (times)	<2	218	68.8%	101	34.7%	72.58	0.000
	2–5	96	30.3%	176	60.5%		
	>5	3	0.9%	14	4.8%		
Interval between the current pregnancy and the last CS (years)	<2	20	6.3%	46	15.8%	15.26	0.002
	2–5	154	48.6%	131	45.0%		
	6–10	108	34.1%	92	31.6%		
	>10	35	11.0%	22	7.6%		
Indications for the previous CS	Elective	262	82.6%	267	91.8%	11.12	0.001
	Postlabor	55	17.4%	24	8.2%		
Hospital grade	Rural hospital	252	79.5%	264	90.7%	14.89	0.000
	University hospital	65	20.5%	27	9.3%		
History of abortion after CS	No	190	59.9%	116	39.9%	24.457	0.000
	Yes	127	40.1%	175	60.1%		
Uterine position	Anteflexion	254	80.1%	174	59.8%	30.096	0.000
	Retroflexion	63	19.9%	117	40.2%		

CS, cesarean section.

### Multivariate logistic regression analysis

Variables that were significant in the univariate analysis were included as independent variables in a binary logistic regression analysis. From the multivariate logistic regression model (Table 3),we can conclude that maternal age older than 35 years, gravidity higher than 3 (especially gravidity higher than 5), more than two induced abortions (especially more than five abortions), an interval between the current CSP and the last CS of less than 5 years (especially less than 2 years), history of CS performed at a rural community hospital, history of induced abortion after CS, and retroposition of uterus were possible independent risk factors for CSP.

**Table 3:** Multivariate logistic regression analysis of factors related to CSP

Variable	Reference group	B	SE	Wald	*P*	OR	95% CI
Maternal age	<35	0.579	0.238	5.915	0.015	1.785	1.119–2.848
Gravidity	<3			16.084	0.000		
3–5		0.814	0.317	6.586	0.010	2.256	1.212–4.200
>5		1.649	0.416	15.698	0.000	5.201	2.301–11.758
Abortion	<2			18.715	0.000		
2–5		0.942	0.233	16.397	0.000	2.566	1.626–4.049
>5		1.891	0.718	6.935	0.008	6.629	1.622–27.089
Interval between the current pregnancy and the last CS	>10			24.205	0.000		
<2		2.157	0.479	20.252	0.000	8.645	3.379–22.116
2–5		1.089	0.383	8.065	0.005	2.970	1.401–6.295
6–10		0.624	0.373	2.794	0.095	1.866	0.898–3.876
Hospital grade	University hospital	0.681	0.285	5.724	0.017	1.976	1.131–3.452
History of abortion after CS	No	0.774	0.191	16.412	0.000	2.168	1.491–3.153
Uterine position	Anteflexion	0.942	0.210	20.054	0.000	2.564	1.698–3.872
Constant		−3.803	0.529	51.715	0.000	0.022	

CS, cesarean section.

## Discussion

In our study, we concluded that there were no unique early clinical features of CSP or similar symptoms of impending abortion, such as vaginal bleeding with or without abdominal pain. In addition, CSP was easily misdiagnosed as early pregnancy during clinical treatment, which often led to blind induced abortion, thus causing uncontrolled hemorrhage. Therefore, it is particularly important for women with a history of menopause and CS to receive early abdominal ultrasound or transvaginal ultrasound during early pregnancy to eliminate CSP.

In this study, we found that a maternal age older than 35 years was an independent risk factor for CSP. There are few similar reports in other studies. The possible reasons may be that with the release of the two‐child policy in China, increasing number women of advanced reproductive age plan to have more children. The pelvises of elderly mothers are more fixed; thus, vaginal delivery is likely to be more difficult for them, which inevitably leads to more CS procedures, thus affecting the healing of the endometrial healing and associated scar.^8^ Besides, before the two‐child policy was introduced, maternal age also affected the rate of induced abortion. The woman who had older maternal age was more tend to choose induced abortion to terminate pregnancy. What is more, maternal age itself may also affect endometrial healing,^9^ unfortunately, although maternal age may be directly associated with previous pregnancy, our study did not include analysis maternal age when the last pregnancy before pregnancy of current study between case group and control group.

At present, it is still unclear whether there is a direct correlation between the incidence of CSP and the number of CS procedures. Jurkovic *et al*.^10^reported that 72% of their CSP patients had undergone multiple (≥2) CS procedures, and they believed that multiple CS procedures led to poor healing of the uterine incision, which was a high risk factor for CSP. The possible pathophysiology is that the normal healing process of the isthmic wall is disrupted by repeated trauma. In addition, poor vascularization in the scar prevents healing.^8^ In our study, we found that 41.49% (121 of 291) of CSP patients had undergone two or more previous CS procedures, while 25.81% (82 of 291) of patients in the control had undergone two or more previous CS procedures (*P* < 0.001). However, we did not find a significant correlation between the number of CS procedures and CSP in the multivariate analysis, consist with the findings of other studies.^11^, ^12^, ^13^ The influence of a previous CS might be masked by other factors that were more influential or by our limited sample size.

Gravidity, parity and the number of induced abortions have been reported to be independent risk factors for CSP.^11^, ^12^ Luo *et al*.^12^found a higher gravidity and frequency of induced abortions in the CSP group than in the control group, but there were no significant differences in the multivariate logistic regression model. However, in our study, we found that both were independent risk factors for CSP. The novelty of our article is that our study was the largest study performed to date in which the history of induced abortion after CS was found to be an independent risk factor for CSP. Multiple pregnancies may result in multiple induced abortions, which injure the endometrium or muscular layer of the uterus, especially when multiple induced abortions are performed after CS, increasing the damage to the integrity of the anterior uterine wall. Luo *et al*.^12^also reported that short intervals between the present and the last pregnancy were correlated with a higher risk of CSP. Qian *et al*.^14^found that a previous CS in a hospital is probably a risk factor for repeated CSP. In accordance with their results, we found that an interval between the current pregnancy and the last CS of less than 5 years, especially less than 2 years, and a history of CS performed at a rural hospital were independent risk factors for CSP. In our study, the shortest interval between the current pregnancy and the last CS was 4 months. A hysterectomy was performed in this woman because of severe placental implantation, which led to uterine rupture and massive bleeding. In China, medical resources in rural hospitals and urban hospitals are very different, and urban hospitals are better equipped and have more qualified gynecologists than rural hospitals.^15^, ^16^ Advanced equipment, experienced gynecologists and excellent suture techniques are detrimental to wound healing,^14^, ^17^ which may be one possible explanation why a history of CS performed at a rural community hospital was an independent risk factor for CSP. But the result is likely to apply only to developing countries.

In recent years, some scholars have proposed the theory of uterine incision defects, which are also called cesarean scar defects. One possible mechanism is that CS itself causes endometrial injury, the incision edges of the uterine are misaligned, or the incision is infected, all of which can contribute to poor wound healing, thus forming cesarean scar defects in the anterior wall of the lower uterine segment. The fertilized egg can easily implant here; if the endometrial stroma deciduates insufficiently or is unable to deciduate, the trophoblast cells of a pregnancy will invade the myometrium directly, even though the uterine wall.^7^, ^15^ Cesarean scar defects have been shown to be associated with various gynecological and obstetric problems, such as uterine rupture and ectopic CSP.^6^, ^18^ There have been a large number of reports about the risk factors for incomplete healing of the uterine incision after CS.^8^, ^17^, ^19^, ^20^, ^21^, ^22^ Consistent with many reports in the literature,^8^, ^9^, ^22^ Chen *et al*.^17^found that after CS, more patients in the post cesarean scar defects (PCSDs) group had retroposition of the uterus. Our study also found that retroposition of the uterus was an independent risk factor for CSP. A possible mechanism is that the segment in the retroflexed uterus may bear increased mechanical tension, which reduces the blood perfusion and oxygenation at the cesarean incision site. In our study, we classified the indications for CS into two categories: elective CS and postlabor cesarean, in which CS is performed after labor. Cephalopelvic disproportion, fetal distress and labor stagnation are the main indications for postlabor CS, while breech presentation, nonmedical factors, abnormal amniotic fluid, and pregnancy complications are the most common indications for elective CS. Shi *et al*.^11^suggested that if elective CS is always performed before the first stage of labor and in an undeveloped lower uterine segment, the cesarean incision site might be imprecise, thus affecting wound healing. Chen *et al*.^17^showed that elective CS was also a risk factor for PCSDs. However, Osser *et al*.^9^ reported that performing CS during advanced labor was associated with an increased risk of incomplete healing of the uterine incision. PCSDs in cases of CS performed during the second stage of labor may be a result of an incision made through the cervical tissue, because during the physiological process of cervical effacement, the cervix becomes incorporated into the lower uterine segment.^19^ Therefore, if a CS is necessary, it would be better to performed CS during the first stage of labor rather than during the second stage of labor. However, we found that there was only statistical significance in the univariate analysis in our study. A history of multiple CS procedures was significantly associated with cesarean scar defects, as reported by many other scholars.^8^, ^9^, ^22^, ^23^, ^24^, ^25^ The possible reason may be that a preexisting CS scar can negatively influence the healing of a new uterine incision due to decreased vascular perfusion and oxygenation in the scar tissue.^26^, ^27^ Antila‐Langsjo *et al*.^23^ found that gestational diabetes mellitus (GDM) increased the risk of incomplete healing of the uterine incision and suggested that diabetes affects incisional healing; thus, this negative effect may be true for GDM as well. However, we only identified a few patients with GDM in the CSP group; thus, we did not include this factor in our study. Other scholars have found that infection, postoperative anemia, and single‐layer uterine closure might contribute to the occurrence of cesarean scar defects.^17^, ^23^, ^28^ Unfortunately, we were notable to collect this information. In addition, Hayakawa *et al*.^25^ found that multiple pregnancies also increased the risk of cesarean scar defects. According to the above literature and our results, we believe that there are similarities between the risk factors for incomplete healing of cesarean scar defects and the risk factors for CSP. The theory of a uterine incision defect may be reasonable, but our report lacked direct evidence for this phenomenon; we could not collect any data regarding if women with CSP had cesarean scar defects after their previous CS procedures. Besides, we chose patients with full‐term pregnancy as the control group, and cases of spontaneous abortion and induced interruption in early gestational age was excluded unintentionally. The one reason is that because our hospital in China belongs to the third class a hospital and the research object of this study is inpatients, early normal spontaneous abortion or induced abortion termination of pregnancy was treated in other basic hospitals or outpatient. Generally, the patients transferred to our hospital are more likely to be suspected of CSP in the outpatient department, or the patients with blind treatment misdiagnosed as normal early intrauterine pregnancy in primary hospitals. However, such choices could cause differences of maternal age, gravidity, parity, previous CS times and induced abortion times and other factors between two groups. As mentioned above, maternal age may affect spontaneous abortion rate and CS rate. So, these risk factors we indicated in this study may be only confounding due to our control choice. Unfortunately, we cannot accurately estimate the effect of such control choices on every risk factor we studied now, which is where we need to improve. Therefore, a prospective study needs to be conducted in the future.

In conclusion, CSP is a product of CS and is associated with multiple factors. Once it is diagnosed, early termination of pregnancy is extremely important to avoid serious complications. Women of child‐bearing age should have planned pregnancies, avoid multiple abortions, especially multiple induced abortions after CS, avoid short intervals between the current and the last CS and not undergo CS without medical indications. Further research is needed to evaluate the relationship between cesarean scar defects and CSP. Detection of cesarean scar defects may be helpful in identifying women at risk of CSP in the future.

## Disclosure

None declared.
